# Cardiac tyrosine hydroxylase activation and MB-COMT in dyskinetic monkeys

**DOI:** 10.1038/s41598-021-99237-5

**Published:** 2021-10-06

**Authors:** Lorena Cuenca-Bermejo, Pilar Almela, Pablo Gallo-Soljancic, José E. Yuste, Vicente de Pablos, Víctor Bautista-Hernández, Emiliano Fernández-Villalba, María-Luisa Laorden, María-Trinidad Herrero

**Affiliations:** 1 Clinical & Experimental Neuroscience (NICE), Institute for Bio-Health Research of Murcia (IMIB), Institute for Aging Research (IUIE), School of Medicine, Campus Mare Nostrum, University of Murcia, 30100 Murcia, Spain; 2 Department of Pharmacology, School of Medicine, Campus Mare Nostrum, University of Murcia, Institute for Bio-Health Research of Murcia (IMIB), 30100 Murcia, Spain; 3 Department of Cardiovascular Surgery, Complejo Hospitalario Universitario A Coruña (CHAUC), La Coruña, Spain

**Keywords:** Neuroscience, Cardiology

## Abstract

The impact of age-associated disorders is increasing as the life expectancy of the population increments. Cardiovascular diseases and neurodegenerative disorders, such as Parkinson’s disease, have the highest social and economic burden and increasing evidence show interrelations between them. Particularly, dysfunction of the cardiovascular nervous system is part of the dysautonomic symptoms of Parkinson’s disease, although more studies are needed to elucidate the role of cardiac function on it. We analyzed the dopaminergic system in the nigrostriatal pathway of Parkinsonian and dyskinetic monkeys and the expression of some key proteins in the metabolism and synthesis of catecholamines in the heart: total and phosphorylated (phospho) tyrosine hydroxylase (TH), and membrane (MB) and soluble (S) isoforms of catechol-O-methyl transferase (COMT). The dopaminergic system was significantly depleted in all MPTP-intoxicated monkeys. MPTP- and MPTP + L-DOPA-treated animals also showed a decrease in total TH expression in both right (RV) and left ventricle (LV). We found a significant increase of phospho-TH in both groups (MPTP and MPTP + L-DOPA) in the LV, while this increase was only observed in MPTP-treated monkeys in the RV. MB-COMT analysis showed a very significant increase of this isoform in the LV of MPTP- and MPTP + L-DOPA-treated animals, with no significant differences in S-COMT levels. These data suggest that MB-COMT is the main isoform implicated in the cardiac noradrenergic changes observed after MPTP treatment, suggesting an increase in noradrenaline (NA) metabolism. Moreover, the increase of TH activity indicates that cardiac noradrenergic neurons still respond despite MPTP treatment.

## Introduction

Parkinson's disease (PD) is the second most diagnosed neurodegenerative disorder clinically classified as a movement disorder. The selective loss of dopaminergic cell bodies in the Substantia Nigra pars compacta (SNpc) and their striatal terminals promote a reduction in dopamine levels, producing the motor alterations^1^. Non-motors symptoms often precede and/or accompany PD onset and progression, but the underlying pathological alterations in the autonomic pathways are not fully understood. Dysfunction of the cardiovascular autonomic nervous system is common in PD, undermining quality of life and contributing to a higher mortality^2^. Studies from cardiac sympathetic neuroimaging indicate that some of the mechanisms involved in PD dysautonomia could be the loss of post-ganglionic noradrenergic nerves^1^ and the severe myocardial noradrenaline (NA) depletion^3^. Moreover, noradrenergic sympathetic nervous system alterations are evidenced in the heart^4^. Particularly, myocardial tissue obtained from PD patients showed decreased tyrosine hydroxylase positive (TH+) neurons, thus indicating cardiac sympathetic denervation^5^. In addition, right ventricular (RV) and left ventricular (LV) myocardium have been reported to differ in their pathophysiological response to several factors^6^.

There is not an experimental model that represents all the manifestation of PD, but intoxication with MPTP is well characterized to provoke nigrostriatal neurotoxicity and cell death, inducing Parkinsonism in humans^7^ and in monkeys^8^. When MPTP is administered to nonhuman primates, they show a bilateral Parkinsonian syndrome with behavioural and neuroanatomical similarities to human condition^9^.

However, the mechanisms of MPTP toxicity to peripheral catecholaminergic system are not very well understood. It has been reported that, in mice, MPTP reduces myocardial NA concentrations^10^ and NA transporter density in their hearts^11^. Additionally, there are other cardiac potential abnormalities such as increased vesicular permeability^12^ or the decrease in NA turnover^13^.

The aim of our study was to identify the functional abnormalities that contribute to myocardial noradrenergic deficiency in PD. For this, we analyzed the expression and activation of TH, as the rate-limiting enzyme in catecholamine biosynthesis, and the expression of membrane (MB) and soluble (S) isoforms of catechol-O-methyl transferase (COMT), an enzyme that metabolizes NA and L-DOPA, in MPTP or MPTP + L-DOPA-treated monkeys.

## Methods

All methods were carried out in accordance with relevant guidelines and regulations.

### Animals

In this study, we have used nonhuman primates for two reasons: (1) their genetic and anatomo-physiological similarities with humans are greater than those of other MPTP rodent models such as rats and mice, and (2) this model is highly qualified to research and understand neurodegenerative processes in PD^14^. The 12 adult male monkeys (*Macaca fascicularis*, 4–6 kg, 4–5 years old, R.C. Hartelust BV, Netherlands) were randomly divided into three groups: (1) control (n = 4); (2) MPTP (n = 4); and, (3) MPTP + L-DOPA (n = 4). Control group animals remained untreated and intoxication, and treatment of groups 2 and 3 are detailed below. All the animals lived individually in cages with dimensions following primates housing regulations. They were placed under controlled conditions of: humidity (50% ± 5%), temperature (24 ± 1 °C), light (12:12 light:dark cycle) and food (Masuri primate diet; Scientific Dietary Services, UK). Water and fresh fruit were available ad libitum. Qualified personnel in animal health care was in charge of monitoring the monkeys’ welfare throughout the study.

All the studies were done in accordance with the European Convention for the protection of Vertebrate Animals used for Experimental and the Council of Europe (no. 123, June 15th, 2006) and with The Code of Ethics of the EU Directive 2010/63/EU. All experimental procedures were approved by the Ethics Committee for Animal Experimentation of the University of Murcia. The study was carried out in compliance with the ARRIVE guidelines.

### Induction of Parkinsonism

Animals from MPTP and MPTP + L-DOPA groups were intoxicated with MPTP (Sigma-Aldrich, St. Louis, USA). MPTP was dissolved in saline and was administered intravenously, one injection (0.3 mg/kg) every 2 weeks for 7 months. At the end of the experiment, animals received a cumulative dose of 7.0 ± 0.2 mg/kg, which has been described as a reproducible intoxication regimen that leads to the first appearance of Parkinsonian clinical signs^15^.

### DOPA treatment

When the monkeys included in the MPTP + L-DOPA group reached a stable parkinsonism, they were treated with Madopar^®^ (Roche, 100 mg/kg) until dyskinesias were developed^13^. The administration regimen consisted in 4 months of daily oral administration of Madopar^®^ + Benserazide (25 mg/kg). Then, the intensity of the dyskinesias was evaluated, every 30 min of a total of 180 min, for each segment of the body (face, neck, trunk, arms and legs), using a dyskinesia disability scale.

### Tissue preparation for post-mortem analyses

The animals were sacrificed 4 h after the last L-DOPA administration by an overdose of sodium pentobarbital (150 mg/kg, intravenous). Brains were fixated by immersion in a 4% paraformaldehyde solution (in 0.01 M phosphate buffered saline, PBS) for 5 days and subsequently placed in a 30% sucrose solution in PBS. Brains were cut coronally along the rostral axis (40 µm-thick) with a freezing microtome (Leica, Germany), collected in 0.125 M PBS + 0.05% sodium azide, and stored at − 20 °C for further free-floating immunohistochemistry. Each heart was dissected and cut longitudinally to obtain LV and RV, which were immediately frozen with dry ice and stored at − 80 °C for further analyses.

### Immunohistochemical techniques

Brain slices that contained the anterior striatum were used for TH and dopamine transporter (DAT) immunohistochemistry, while ventral mesencephalon sections (III cranial pair exit) were immunostained for TH. Sections were incubated with 0.02% hydrogen peroxide (in PBS) for endogenous peroxidase blocking and then incubated for 30 min with normal goat serum (5% in 0.2% Triton X-100, Sigma-Aldrich). After, the sections were incubated in the same solution overnight at 4 °C with the primary antibodies anti-TH (mouse monoclonal, 1:1000; Chemicon, CA, USA) and anti-DAT (rat monoclonal, 1:1000; Chemicon). Then, sections were washed with PBS and incubated with the secondary antibodies (30 min): biotinylated goat anti-mouse (1:200 in PBS; Agilent, CA, USA) and biotinylated rabbit anti-rat (1:200 in PBS; Chemicon). They were incubated with the Vector avidin–biotin complex (1:200, Vectastain Elite ABC kit, Vector Laboratories, USA) and DAB substrate kit (Vector Laboratories) was used to stain the sections. Additionally, mesencephalon slices were selected and stained for thionine. All the slices were washed and mounted on gelatin-coated slides and coverslipped (DPX, Sigma-Aldrich).

TH and DAT optical density was performed in the striatum of all animals at the rostral level (anterior commissure + 2 mm) including the head of the caudate and putamen. The relative optical densities of TH+ and DAT+ fibers were quantified using computer-assisted image analysis (ImageJ 1.41, National Institutes of Health, USA) in four different areas: dorsolateral (DL), dorsomedial (DM), ventrolateral (VL) and ventromedial (VM). Seven sections from rostro-caudal levels, equally spaced (intervals of 2.4 mm), were examined for each monkey. Three sections were more rostral and 4 sections more caudal to the level where the anterior commissure crosses the midline.

The number of TH+ neurons was quantified in the ventral mesencephalon by the optical fractionator in the SNpc and Ventral Tegmental Area (VTA)^16^.

All images were taken in black and white 8-bit monochrome using with a digital camera (AxioCam HRc, Zeiss, Germany) coupled to an interactive computer system consisting of a Zeiss Axioskop optical microscope (Oberkochen, Germany).

### Western blot analysis

Western blot was performed for total TH, phosphorylated (phospho) TH, and MB- and S-COMT in the RV and LV. Samples were placed in homogenization buffer [PBS, 2% sodium dodecylsulfate (SDS) plus protease inhibitors (Roche, Germany) and phosphatase inhibitors Cocktail Set (Calbiochem, Germany)], homogenized (50 s) and centrifuged (6000*g*, 20 min, 4 °C). Total protein concentrations were determined spectrophotometrically using the bicinchoninic acid method^17^. The optimum amount of protein to be loaded was determined in preliminary experiments by loading gels with increasing protein contents (25–100 mg) from samples of each experimental group. 50 μg of protein/lane from each sample were loaded on a 10% SDS–polyacrylamide gel (SDS–PAGE), electrophoresed, and transferred onto a PVDF membrane using a Mini Trans-Blot Electrophoresis Transfer Cell (Bio-Rad Laboratories, CA, USA). Membranes were blocked with 1% bovine serum albumin (BSA) in tris buffer saline tween (TBST: 10 mmol/L Tris–HCl, pH 7.6, 150 mmol/L NaCl, and 0.05% Tween 20). Blots were incubated at 4 °C with primary antibodies: polyclonal anti-TH; 1:1000; AB152, Chemicon), polyclonal anti-P-TH Ser-40 (1:500; AB5935, Chemicon), monoclonal anti-COMT (1:5000; AB5873, Chemicon), in TBST with BSA. After TBST washes, the membranes were incubated (1 h, RT) with the peroxidase-labelled secondary antibodies: anti-rabbit sc-2004 for total and phosphorylated TH (1:2500), and anti-mouse sc-2005 for COMT (1:5000). Blots were washed and immunoreactivity was detected with a chemiluminescent/chemifluorescent detection system (ECL Plus, GE Healthcare, LittleChalfont, Buckinghamshire, UK) and visualized by a Typhoon 9410 variable mode Imager (GE Healthcare). Anti β-actin (Cell Signaling, 45 kDa) was used as loading control. The ratio total TH/β-actin, phospho-TH/β-actin, MB-COMT/β-actin and S-COMT/β-actin was plotted and analysed.

Quantification of immunoreactivity corresponding to total TH (60 kDa), P-TH Ser-40 (60 kDa), MB-COMT and S-COMT (30 and 25 kDa, respectively) bands was carried out by densitometry (AlphaImager, Nucliber, Madrid).

Some examples of the original gels from total TH in the right and left ventricle, P-TH Ser-40 in the right and left ventricle, MB-COMT and S-COMT in the right and left ventricle, MB-COMT and S-COMT in the left ventricle and Beta-actin in the left ventricle are shown in Supplementary Figure 1.

### Statistical analysis

Data was collected and analyzed blindly. Differences in TH+ neurons were analysed using one-way repeated measures analyses of variance (ANOVA) followed by the Bonferroni *post-hoc* test. Comparison of TH+ and DAT+ stainings among striatal subregions in MPTP-treated monkeys as well as total TH and phospho-TH, S-COMT and MB-COMT analysis was performed using one-way ANOVA followed by Newman-Keuls post-hoc test. Two-sided *p* values of less than 0.05 were considered significant. Statistical analyses were done with GraphPad Prism software (GraphPad Prism version 5.0 software for Windows; California, USA).

## Results

### Brain tissue

We performed TH and DAT immunostaining in two relevant brain areas in PD (striatum and ventral mesencephalon) in order to confirm that MPTP induced the histopathological model of the disease.

### Dopaminergic neuronal loss in the ventral mesencephalon

**Figure 1:**
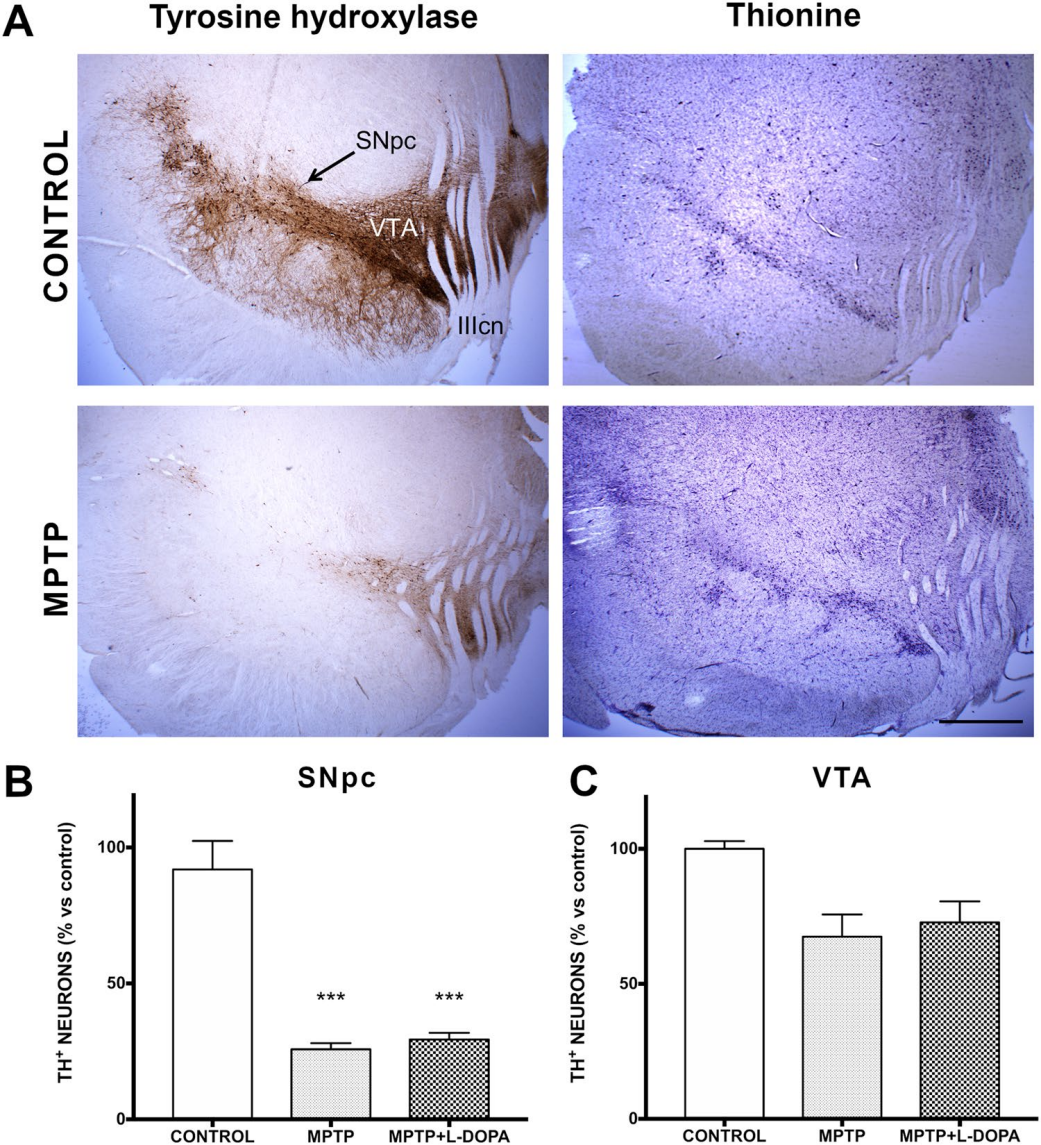
Evaluation of dopaminergic neuronal loss in the ventral mesencephalon. (**A**) Representative micrographs of TH immunolabeling and thionine staining (scale bar = 1 mm; *SNpc* Substantia Nigra pars compacta, *VTA* ventral tegmental area, *IIIcn* III cranial nerve). (**B**, **C**) Scatter plots representing the number of TH + ir cells (% versus control, mean ± SD) in the SNpc (**B**) and in the VTA (**C**). Data were compared by one-way ANOVA (SNpc: F_(2,9)_ = 30.15, *P* = 0.0007; VTA: F_(2,9)_ = 4.213, *P* = 0.072) followed by the Bonferroni post-hoc test. Values are represented as mean ± standard deviation. ***Indicate significant differences with *P* < 0.001 compared to the control group (n = 4 biological replicates and 7 technical replicates/animal).

Representative micrographs of TH immunolabeling and thionine staining in SNpc and VTA are shown in Fig. 1A. The stereological count of TH+ neurons in the SNpc revealed a very significant reduction (*P* < 0.001) in comparison to control animals in the groups of MPTP and MPTP + L-DOPA monkeys, in which, approximately, only 26 and 29% of the TH+ neurons survive, respectively (Fig. 1B). In the VTA, we also observed a very significant reduction in the number of TH+ cells in both groups (*P* < 0.001; Fig. 1C).

#### Assessment of terminal densities in the striatum

**Figure 2:**
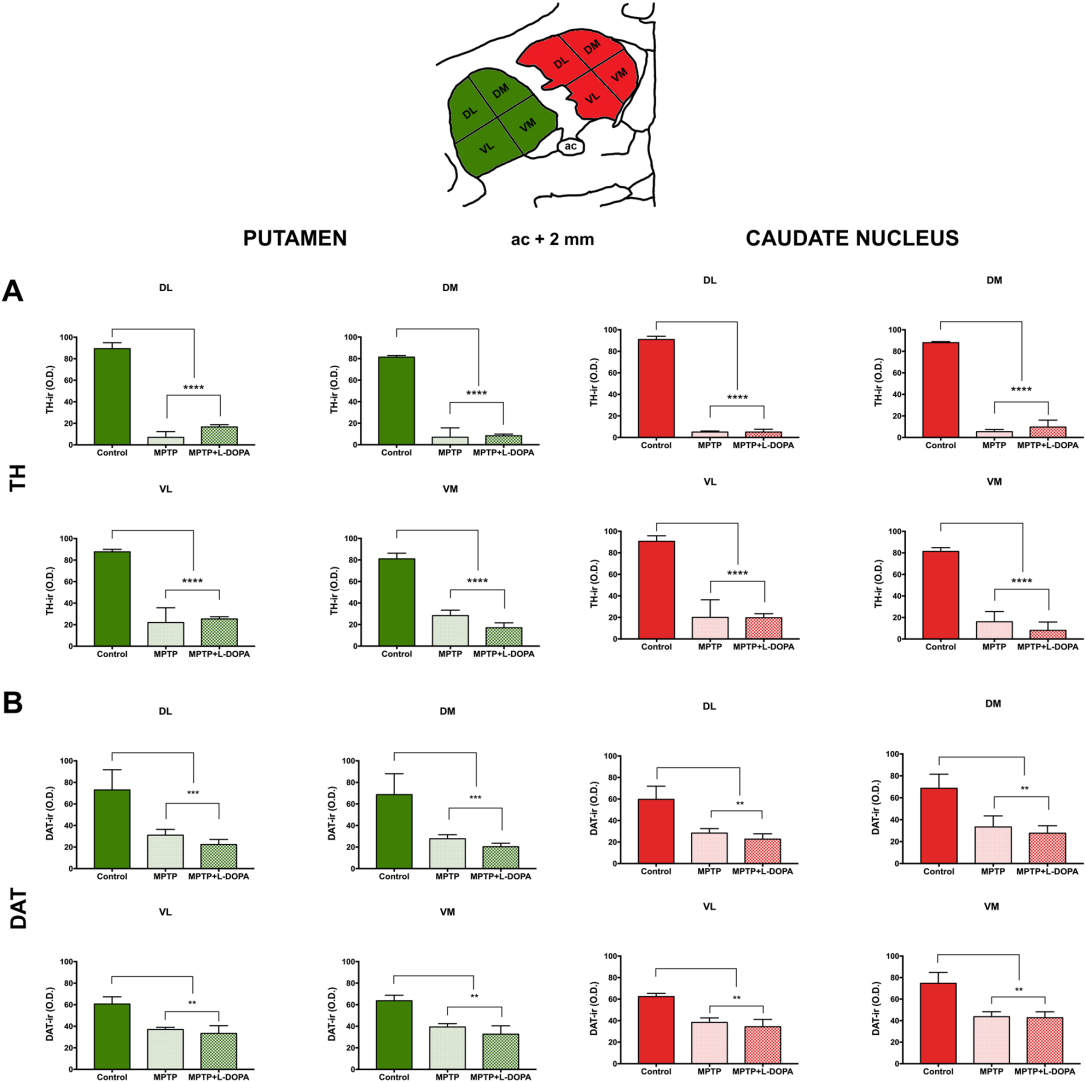
Striatal TH + ir fibers distribution and DAT expression in the anterior striatum (anterior commissure =  + 2 mm). (**A**) Quantification of TH optical density in the putamen (left, green) and caudate nucleus (right, red). (**B**) Quantification of DAT optical density in the putamen (left, green) and caudate nucleus (right, red). Data were compared by one-way ANOVA (Putamen. TH: DL, F_(2,9)_ = 273.7, *P* < 0.0001; DM F_(2,9)_ = 195.4, *P* < 0.0001; VL, F_(2,9)_ = 40.42, *P* < 0.0003; VM, F_(2,9)_ = 139.2, *P* < 0.0001. DAT: DL, F_(2,9)_ = 42.50, *P* = 0.0003; DM, 42.41, *P* = 0.0003; VL, F_(2,9)_ = 26.30, *P* = 0.0011; VM, F_(2,9)_ = 34.94, *P* = 0.0005. Caudate nucleus. TH: DL, F_(2,9)_ = 1047, *P* < 0.0001; DM F_(2,9)_ = 276, *P* < 0.0001; VL, F_(2,9)_ = 43.05, *P* = 0.0003; VM, F_(2,9)_ = 64.95, *P* < 0.0001. DAT: DL, F_(2,9)_ = 18.07, *P* = 0.0029; DM, 15.27, *P* = 0.0044; VL, F_(2,9)_ = 26.11, *P* = 0.0011; VM, F_(2,9)_ = 17.66, *P* = 0.0031) followed by Bonferroni post-hoc test. Values are represented as mean ± standard deviation. Symbols indicate significant differences compared with the control group: **P < 0.01 and ***P < 0.001. (n = 4 biological replicates and 7 technical replicates/animal). *Ac* anterior commissure, *DAT* dopamine transporter, *DL* dorsolateral, *DM* dorsomedial, *VL* ventrolateral, *VM* ventromedial, *TH* tyrosine hydroxylase.

A reduction in the level of immunoreactivity for both dopaminergic markers (TH and DAT) was observed in the anterior striatum of all MPTP and MPTP + L-DOPA-treated monkeys (Fig. 2). TH immunostaining was very significantly decreased in all MPTP and MPTP + L-DOPA groups, both in the putamen (dorsomedial: *P* < 0.001; dorsolateral: *P* < 0.001; ventromedial: *P* < 0.001; *P* < 0.001) and in the caudate (dorsomedial: *P* < 0.001; dorsolateral: *P* < 0.001; ventromedial: *P* < 0.001; ventrolateral: *P* < 0.001) (Fig. 2A). Similar results were obtained in the DAT + of the putamen (dorsomedial: *P* < 0.001; dorsolateral: *P* < 0.001; ventromedial: *P* < 0.001; *P* < 0.001) and in the caudate nucleus (dorsomedial: *P* < 0.001; dorsolateral: *P* < 0.001; ventromedial: *P* < 0.001; ventrolateral: *P* < 0.001) (Fig. 2B).

### Heart tissue

#### Effects of Parkinsonism and L-DOPA treatment on total TH and phospho-TH at Ser-40 expression

We have evaluated the heart weight in the different groups studied finding no statistical differences among them (control: 38 ± 01.0 g; MPTP: 36 ± 1.0 g; MPTP + L-DOPA 38 ± 0.1 g). There were also no statistically significant differences when comparing the weights of the different groups in RV (control: 6.50 ± 0.70 g; MPTP: 6.0 ± 0.0 g; MPTP + L-DOPA: 5.66 ± 1.15 g) or LV (control: 14.50 ± 0.70 g; MPTP: 13.67 ± 0.57 g; MPTP + L-DOPA: 14.33 ± 0.57 g).

**Figure 3:**
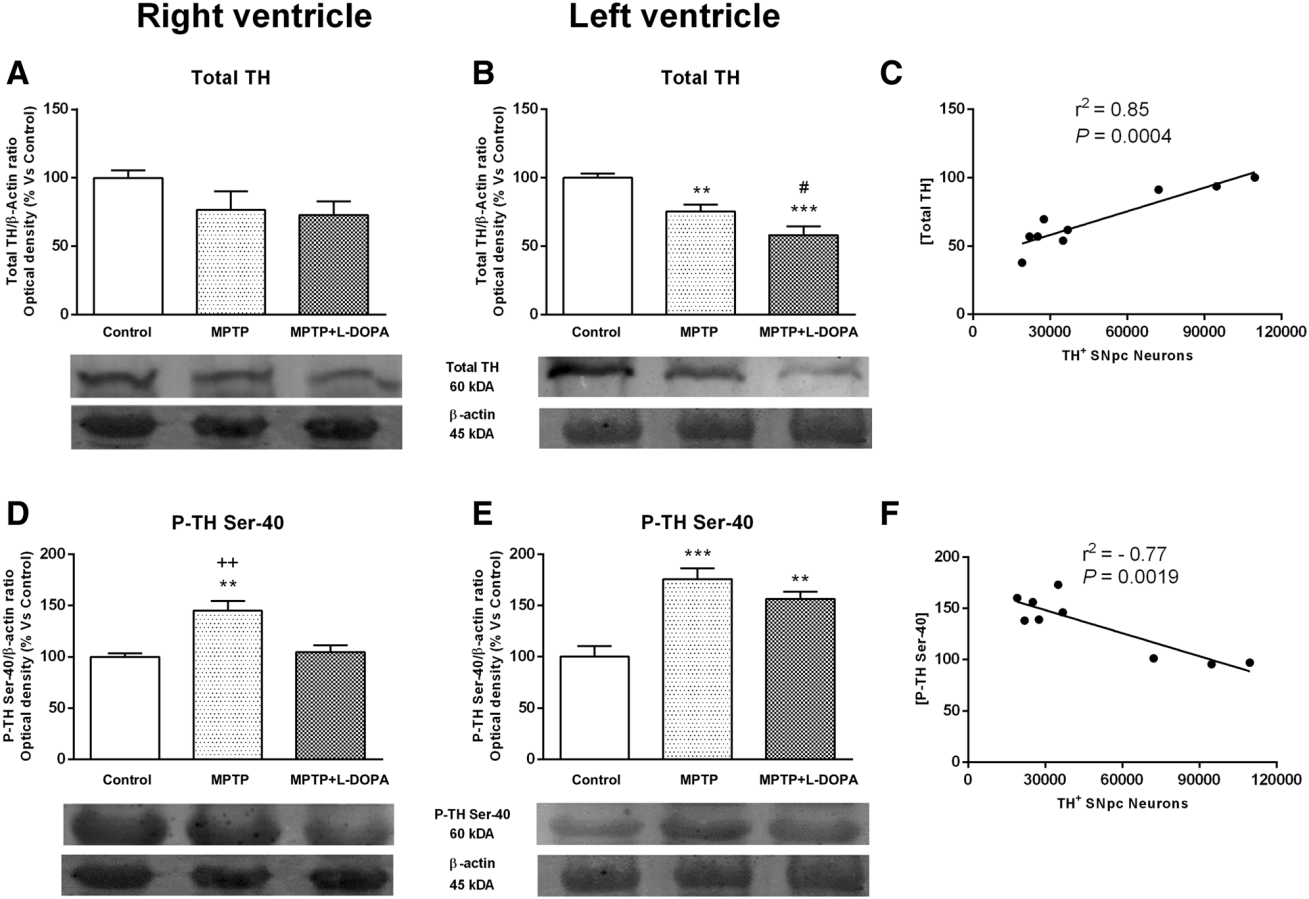
Effects of MPTP and L-DOPA treatment on total TH and phospho-TH at Ser-40 expression in the monkey heart. (**A**, **B**) Total TH/β-actin ratio (optical density, % vs control) in controls, MPTP-treated monkeys and in the MPTP + L-DOPA group. Total TH expression was significantly decreased in the LV in MPTP-treated monkeys and in the MPTP + L-DOPA group versus control group and in the MPTP + L-DOPA group versus MPTP-treated animals. (**C**) Strong correlation between the number of TH + neurons in the SNpc and total TH levels in the heart tissue (r^2^ = 0.85, P = 0.0004). (**D**, **E**) P-TH at Ser-40/β-actin ratio (optical density, % vs control) in controls, MPTP-treated monkeys and in the MPTP + L-DOPA group. TH phosphorylation at Ser-40 was significantly increased in the RV with respect to the other two groups. Besides, in the LV, we found an important increase of phospho-TH at Ser-40 in both groups (MPTP or MPTP + L-DOPA treatment) with respect to controls. (**F**) Strong correlation between phospho-TH and TH+ neurons in the SNpc (r^2^ = 0.77, P = 0.0019). Data were compared by one-way ANOVA (total TH: F_(2,9)_ = 2.063, *P* = 0.1831, RV; F_(2,9)_ = 17.87, *P* = 0.0007, LV. *P*-TH: F_(2,9)_ = 12.77, *P* = 0.0024, RV; F_(2,9)_ = 17.43, *P* = 0.0008, LV) followed by Newman–Keuls post-hoc test. Values are represented as mean ± standard deviation. **P < 0.01, ***P < 0.001 versus control group; ^#^P < 0.05 versus MPTP-treated animals; ^++^P < 0.01 versus MPTP + L-DOPA. *TH* tyrosine hydroxylase, *MPTP* 1-methyl-4-phenyl-1,2,3,6-tetrahydropyridine, *L-DOPA* levodopa, *P* phospho. (n = 4 animals/group and 2 technical replicates/animal).

Total TH expression and TH phosphorylation at Ser-40 in the RV and LV were analysed in control group, MPTP- or MPTP + L-DOPA-treated-animals to determine the impact of Parkinsonism and its treatment on the noradrenergic cardiac pathways. As expected, MPTP-treated animals showed a decrease in total TH expression in both RV and LV versus control group. However, this relationship became significant (*P* < 0.01) only in the LV. L-DOPA administration induced a significant reduction of total TH expression in LV versus control group (*P* < 0.001) and also in MPTP-treated animals (*P* < 0.05) (Fig. 3A,B). Moreover, there was a significant correlation between the number of TH^+^ neurons in the SNpc and total TH levels in the heart tissue from control, MPTP or MTP + L-DOPA groups (r^2^ = 0.85, *P* = 0.0004, Fig. 3C).

In the RV, a significant increase of phospho-TH was observed in MPTP-treated monkeys with respect to the other groups (*P* < 0.01). However, in the LV, we found an important increase of phospho-TH in both groups (MPTP or MPTP + L-DOPA treatment) compared to control animals (*P* < 0.001 and *P* < 0.01, respectively) (Fig. 3D,E), suggesting that L-DOPA treatment could activate a phosphorylation mechanism in TH protein and exert a compensative effect in the LV myocardium. Moreover, we observed that there was an inverse significant correlation between TH^+^ neurons in the SNpc and phospho-TH levels in the heart tissue (r^2^ = − 0.77, *P* = 0.0019, Fig. 3F), reinforcing the idea of a close relationship between central and peripheral catecholaminergic changes in MPTP-treated monkeys^18,19^.

#### COMT activation after chronic MPTP and L-DOPA treatment

**Figure 4:**
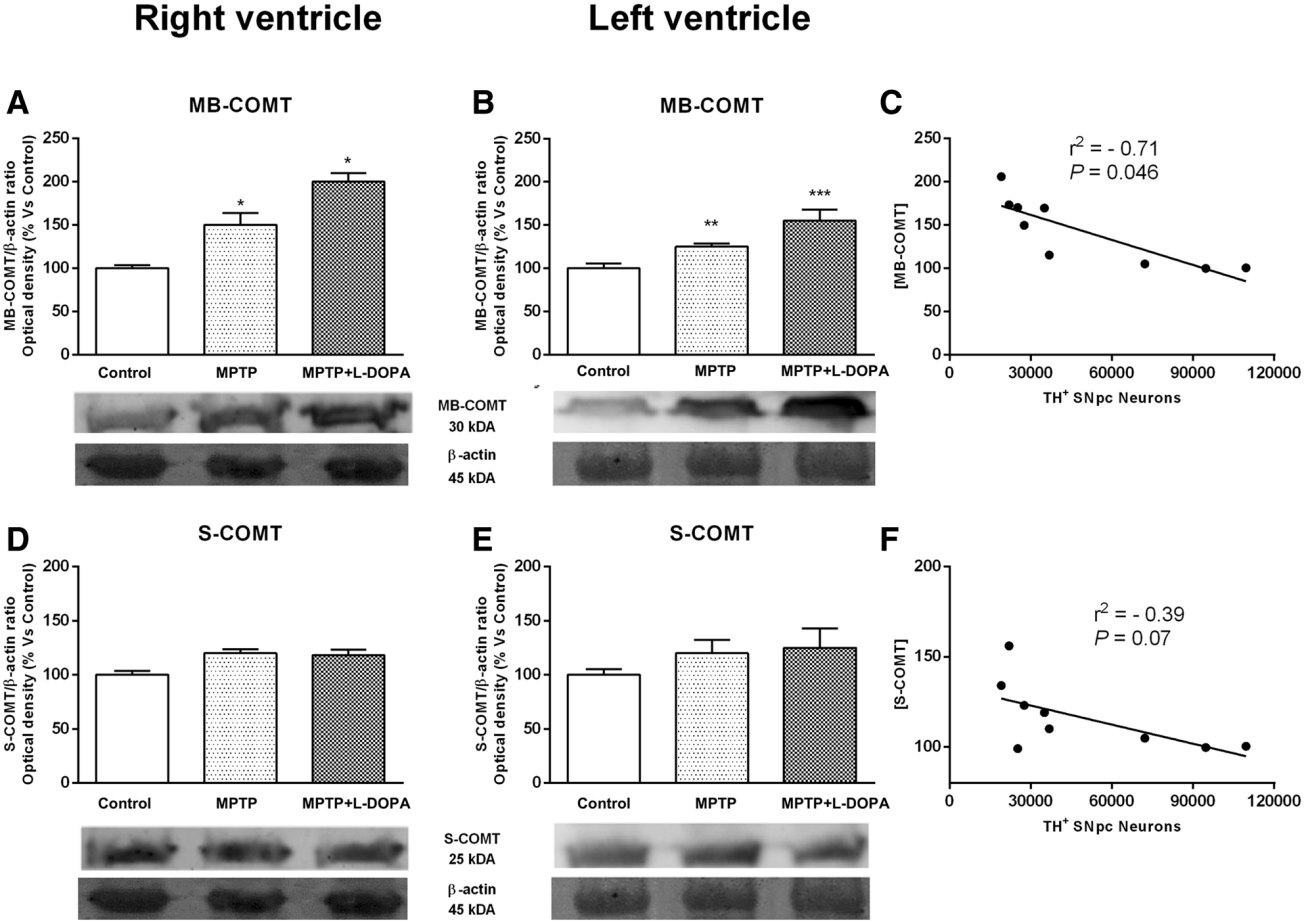
Effects of MPTP and L-DOPA treatment on COMT in the monkey heart. (**A**, **B**) MB-COMT/β-actin ratio (optical density, % vs control) in controls, MPTP-treated monkeys and in the MPTP + L-DOPA group. MB-COMT expression was increased in the RV of MPTP- and MPTP + L-DOPA-treated animals compared to control group. (**C**) Significant correlation between MB-COMT and TH+ neurons in the SNpc (r^2^ = 0.71, P = 0.0046). (**D**, **E**) S-COMT/β-actin ratio (optical density, % vs control) in controls, MPTP-treated monkeys and in the MPTP + L-DOPA group. The quantitative analysis showed no differences between groups. (**F**) No correlation between S-COMT and TH+ neurons in the SNpc was observed. Data were compared by one-way ANOVA (MB-COMT: F_(2,9)_ = 14.41, *P* = 0.0016, RV; F_(2,9)_ = 11.62, P = 0.0032, LV. S-COMT: F_(2,9)_ = 3.31, *P* = 0.0827, RV; F_(2,9)_ = 1.742, *P* = 0.2293, LV) followed by Newman–Keuls post-hoc test. Values are represented as mean ± standard deviation. (*P < 0.05, **P < 0.01, ***P < 0.001 versus control group). *MB* membrane, *S* soluble, *COMT* catechol-O-methyl transferase, *MPTP* 1-methyl-4-phenyl-1,2,3,6-tetrahydropyridine, *L-DOPA* levodopa. (n = 4 animals/group and 2 technical replicates/animal).

Considering the fact that little or nothing is known about the differences of the physiological roles of S-COMT and MB-COMT in the heart, we found interesting to evaluate in this study both isoforms of this protein. We obtained two bands at approximately 30 and 25 kDa. Based on the sizes and relevant abundance, the 30 kDa bands are presumed to be the membrane subunit MB-COMT in the heart, and the 25 kDa ones correspond to S-COMT. Quantitative analysis showed non-significant changes in S-COMT expression in any of the groups studied (Fig. 4D,E). In contrast, we observed a significant increase in MB-COMT expression in the RV of MPTP- and MPTP + L-DOPA-treated animals compared to the control group (*P* < 0.05). Similar results were observed in the LV, an increase in this protein after MPTP or MPTP + L-DOPA treatment with respect to control animals (*P* < 0.01 and *P* < 0.001, respectively, Fig. 4A,B).

Consistently with our previous findings demonstrating a close relationship between catecholaminergic central and peripheral changes, we also observed an inverse significant correlation between TH^+^ neurons in the SNpc and MB-COMT in the heart tissue (r^2^ = − 0.71, *P* = 0.046, Fig. 4c). In contrast, we did not observe a significant correlation between TH^+^ neurons in the SNpc and S-COMT (r^2^ = − 0.39, *P* = 0.07, Fig. 4F).

## Discussion

PD is a progressive neurodegenerative disorder characterized by the increasing degeneration of dopaminergic neurons in the SNpc, which produces severe motor alterations and non-motor symptoms, including autonomic and cardiovascular dysfunction^1^. Several studies have reported that PD entails profound alterations in cardiac noradrenergic pathways that affect, not only to neuronal loss, but also to other neuronal functions such as NA biosynthesis via TH or NA turnover^13,20^. Importantly, in the present study, we report a strong correlation between neuronal cell death in the SNpc and cardiac sympathetic pathways. This result is consistent with a previous study in monkeys demonstrating that MPTP exposure can induce nigral dopaminergic cell loss in parallel with the degeneration of cardiac TH − ir^18^. In addition, we evaluate MPTP-induced changes in NA synthesis and metabolism, including L-DOPA treatment. The different responses observed in LV and RV to MPTP toxicity suggest that the pattern of cardiac sympathetic dysfunction in PD is not homogeneous in both ventricles as it has been described previously^21^.

The effect of MPTP on the central nervous system has been largely reported by others and us in the literature^13,15^. In the present study, a nigrostriatal degeneration with a marked decrease in the number of striatal TH+ fibers and dopaminergic cells in the SNpc and in the VTA was observed. According to previous studies^22,23^ we found that the striatum of Parkinsonian monkeys chronically treated with L-DOPA exhibited a small number of TH+ fibers, similar to the MPTP-treated group, when compared to control animals. Additionally, DAT optical density was dramatically decreased in the striatum of MPTP and MPTP + L-DOPA groups compared to the control one, both in the putamen and in the caudate nucleus (Supplementary Figure 1).

On the other hand, clinical and pathological studies have provided strong evidence of the involvement of cardiac sympathetic nerves in PD patients^11^. In our study, we also demonstrated a decrease in total TH expression in LV together with an enhancement of TH activity, suggesting an alteration in the cardiac noradrenergic pathway in Parkinsonian monkeys without L-DOPA treatment. According to this result, different studies have suggested the denervation of Parkinsonian heart with a decrease in TH − ir axons in the epicardial layer of LV anterior wall^3^. However, studies reporting functional evidence of cardiac denervation are uncommon. Present results demonstrate different ventricular responses to MPTP toxicity and posterior L-DOPA treatment, being the LV the most sensible. The amount and distribution of sympathetic loss in PD seems to vary among individuals and it has been reported as either diffuse, at times extensively, or following no uniform pattern with maximal loss in the apex and the inferior and lateral walls of the LV^24^. Consistently, we observed a decrease in TH protein expression in monkey cardiac tissue in both ventricles, existing a significant correlation between total TH levels and the number of TH+ neurons in SNpc. These results could help us to understand the interrelation between brain and heart and to consider new therapies to eliminate or reverse the alterations induced in both organs in PD. Concomitantly with the increase in TH expression we have observed an enhancement in TH phosphorylation at Ser-40. The decrease in TH protein level can be explained as a compensatory mechanism of catalytic activity by increased phosphorylation^24^. TH phosphorylated at Ser-40 increased levels in both ventricles accelerated TH activity, thereby stimulating production of neurotransmitter in catecholaminergic terminals^25^. The increase of sympathetic activity observed in this study could be implicated in the increase of heart rate described in MPTP-treated mice^26^. Our results demonstrating an increased TH activity in Parkinsonian monkeys agree with the idea that loss of sympathetic noradrenergic innervation induce abnormalities in residual nerves that are dysfunctional but extant^20^. Importantly, present results, together with a previous study which demonstrated an increased in the NA turnover after MPTP + L-DOPA treatment, support this idea^13^. Together with the increased TH activity, the present study shows for the first time an enhancement of MB-COMT expression in both ventricles in Parkinsonian monkeys before and after L-DOPA treatment without any changes in S-COMT. Moreover, we found a strong correlation between the number of TH+ SNpc neurons and MB-COMT, suggesting that membrane isoform of COMT has an important role in NA metabolism. In this line, a recent study showed that MB-COMT has a higher catalytic activity and affinity but lower capacity towards monoamine substrates than S-COMT^27^. Both COMT isoforms play distinct role: S-COMT has a predominant role when the substrates levels are high and it is involved in the O-methylation and elimination of biologically active, toxic and mainly exogenous catecholamines^28^, while MB-COMT is involved in the termination of catecholaminergic synaptic neurotransmission when there are low concentrations of catecholamines^28^. We have previously demonstrated a decrease of NA levels in the RV and LV before and after L-DOPA treatment^13^, which could induce the enhancement in MB-COMT expression in the RV and LV observed in the present study. Support for this hypothesis has been found in a recent study showing that MB-COMT levels might increase in nervous tissues as a result of its release from disintegrating neurons^29^.

The enhancement in MB-COMT could be responsible for the increase of normetanephrine content and NA turnover showed after MPTP + L-DOPA treatment in a previous study^13^. However, whether L-DOPA is neurotoxic or neuroprotective is still an issue of debate and no studies have directly evaluated the toxic or protective cardiac effects of this drug^30^. The increased levels of MB-COMT observed in our study suggest that drugs that inhibit the degradation of L-DOPA and DA, like COMT inhibitors (tolcapone and entacapone) could improve the cardiac response in PD. Our study also concordats with the use of COMT inhibitors which is currently chosen as a complementary therapy to L-DOPA such as opicapone, a novel third generation long-acting peripheral COMT inhibitor, being a safe and successful clinical choice^31^, even if Zeng and collaborators found no significant changes in brain and liver COMT levels^32^.

In conclusion, our results indicate impairments of NA synthesis and metabolism in Parkinsonism, with enhancement in the expression of MB-COMT. These results support the hypothesis that the residual cardiac innervation is able to respond to different events. These data can contribute to our knowledge about the mechanisms implicated in the cardiovascular dysfunction observed in PD and suggest that COMT inhibitors, with preferential affinity for MB-COMT over S-COMT, could improve the response of cardiac noradrenergic pathway in PD.

## Supplementary Information

Supplementary Figure 1.

## Abbreviations

COMT: Catechol-O-methyltransferase
DA: Dopamine
DAT: Dopamine transporter
L-DOPA: Levodopa
LIDs: L-DOPA-induced dyskinesias
LV: Left ventricle
MPTP: 1-Methyl-4-phenyl-1,2,3,6-tetrahydropyridine
NA: Noradrenaline
NMN: Normetanephrine
PD: Parkinson’s disease
RV: Right ventricle
SNpc: Substantia Nigra pars compacta
TH: Tyrosine hydroxylase
VTA: Ventral tegmental area

## References

[1] Cuenca L, Gil-Martinez AL, Cano-Fernández L, Sánchez-Rodrigo C, Estrada C, Fernández-Villalba E, Herrero MT (2019). Parkinson's disease: A short story of 200 years. Histol. Histopathol..

[2] Bouhaddi M, Vuillier F, Fortrat JO, Cappelle S, Henriet MT, Rumbach L, Regnard J (2004). Impaired cardiovascular autonomic control in newly and long-term-treated patients with Parkinson’s disease: involvement of L-dopa therapy. Auton. Neurosci..

[3] Jain S, Goldstein DS (2012). Cardiovascular dysautonomia in Parkinson disease: From pathophysiology to pathogenesis. Neurobiol. Dis..

[4] Mitsui J, Saito Y, Momose T, Shimizua J, Araia N, Shibaharad J, Ugawa Y, Kanazawa I, Tsuji S, Murayama S (2006). Pathology of the sympathetic nervous system corresponding to the decreased cardiac uptake in 123I-metaiodobenzylguanidine (MIBG) scintigraphy in a patient with Parkinson disease. J. Neurol. Sci..

[5] Orimo S, Uchihara T, Nakamura A, Mori F, Ikeuchi T, Onodera O, Nishizawa M, Ishikawa A, Kakita A, Wakabayashi K, Takahashi H (2008). Cardiac sympathetic denervation in Parkinson’s disease linked to SNCA duplication. Acta Neuropathol..

[6] Friehs I, Cowan DB, Choi YH, Black KM, Barnett R, Bhasin MK, Daly C, Dillon SJ, Libermann TA, McGowan FX, del Nido PJ, Levitsky S, McCully JD (2013). Pressure-overload hypertrophy of the developing heart reveals activation of divergent gene and protein pathways in the left and right ventricular myocardium. Am. J. Physiol. Heart Circ. Physiol..

[7] Langston JW, Ballard P, Tetrud JW, Irwin I (1983). Chronic Parkinsonism in humans due to a product of meperidine-analog synthesis. Science.

[8] Langston JW, Langston EB, Irwin I (1984). MPTP-induced Parkinsonism in human and non-human primates: Clinical and experimental aspects. Acta Neurol. Scand. Suppl..

[9] Pérez-Otaño I, Herrero MT, Luquin MR, Obeso JA, Del Río J (1992). Chronic MPTP treatment reduces substance P and met-enkephalin content in the basal ganglia of the marmoset. Brain Res..

[10] Luthman J, Sundström E (1990). No apparent difference in the effects of 1-methyl-4-phenyl-1,2,3,6-tetrahydropyridine (MPTP) on the sympathetic system in NMRI and C57 BL/6 mice. Toxicol. Lett..

[11] Fujishiro H, Frigerio R, Burnett M, Klos KJ, Josephs KA, DelleDonne A, Parisi JE, Ahlskog JE, Dickson DW (2008). Cardiac sympathetic denervation correlates with clinical and pathologic stages of Parkinson’s disease. Mov. Disord..

[12] Plotegher N, Berti G, Ferrari E, Tessari I, Zanetti M, Lunelli L, Greggio EM, Veronesi M, Girotto S, Dalla Serra M, Perego C, Casella L, Bubacco L (2017). DOPAL derived alpha-synuclein oligomers impair synaptic vesicles physiological function. Sci. Rep..

[13] Almela P, Cuenca-Bermejo L, Yuste JE, Estrada C, de Pablos V, Bautista-Hernández V, Fernández-Villalba E, Laorden ML, Herrero MT (2019). Cardiac noradrenaline turnover and heat shock protein 27 phosphorylation in dyskinetic monkeys. Mov. Disord..

[14] Blesa J, Trigo-Damas I, Del Rey NL, Obeso JA (2018). The use of nonhuman primate models to understand processes in Parkinson's disease. J. Neural. Transm. (Vienna)..

[15] Barcia C, De Pablos V, Bautista-Hernández V, Sánchez-Bahillo A, Fernández-Barreiro A, Poza M, Herrero MT (2004). Measurement of motor disability in MPTP-treated macaques using a telemetry system for estimating circadian motor activity. J. Neurosci. Methods..

[16] West MJ (1999). Stereological methods for estimating the total number of neurons and synapses: Issues of precision and bias. Trends Neurosci..

[17] Wiechelman KJ, Braun RD, Fitpatrick JD (1988). Investigation of the bicinchoninic acid protein assay: Identification of the groups responsible for colour formation. Anal. Biochem..

[18] Carmona-Abellán M, Martínez-Valbuena I, DiCaudo C, Marcilla I, Luquin MR (2019). Cardiac sympathetic innervation in the MPTP non-human primate model of Parkinson disease. Clin. Auton. Res..

[19] Critchley BJ, Isalan M, Mielcarek M (2018). Neuro-cardio mechanisms in Huntington's disease and other neurodegenerative disorders. Front. Physiol..

[20] Goldstein DS, Pekker MJ, Eisenhofer G, Sharabi Y (2019). Computational modelling reveals multiple abnormalities of myocardial noaredenrgic function in Lewy body disease. JCI Insight..

[21] Li ST, Dendi R, Holmes C (2002). Progressive loss of cardiac sympathetic innervation in Parkinson’s disease. Ann. Neurol..

[22] Herrero MT, Augood SJ, Asensi H, Hirsch EC, Agid Y, Obeso JA, Emson PC (1996). Effects of L-DOPA-therapy on dopamine D2 receptor mRNA expression in the striatum of MPTP-intoxicated Parkinsonian monkeys. Mol. Brain Res..

[23] Herrero MT, Levy R, Ruberg M, Luquin MR, Villares J, Guillen J, Faucheux B, Javoy-Agid F, Guridi J, Agid Y, Obeso JA, Hirsch EC (1996). Consequence of nigrostriatal denervation and L-dopa therapy on the expression of glutamic acid decarboxylase messenger RNA in the pallidum. Neurology.

[24] Nakashima A, Ota A, Kaneko YS, Mori K, Nagasaki H, Nagatsu T (2013). A possible pathophysiological role of tyrosine hydroxylase in Parkinson's disease suggested by postmortem brain biochemistry: A contribution for the special 70th birthday symposium in honor of Prof. Peter Riederer. J. Neural. Transm..

[25] Dunkley P, Bobrowskaya L, Graham ME, von Nagy-Felsobuki EI, Dickson PW (2004). Tyrosine hydroxylase phosphorylation: regulation and consequences. J. Neurochem..

[26] Liu X, Wei B, Bi Q, Sun O, Li L, He J, Weng Y, Zhang S, Mao G, Bao Y, Wan S, Shen XZ, Yan J, Shi P (2020). MPTP-induced impairment of cardiovascular function. Neurotox. Res..

[27] Magarkar A, Parkkila P, Viitala T, Lajunen T, Mobarak E, Licari G, Cramariuc O, Vauthey E, Róg T, Bunker A (2018). Membrane bound COMT isoform is an interfacial enzyme: General mechanism and new drug design paradigm. Chem. Commun..

[28] Männistö PT, Kaakkola S (1999). Catechol-O-methyltransferase (COMT): Biochemistry, molecular biology, pharmacology, and clinical efficacy of the new selective COMT inhibitors. Pharmacol. Rev..

[29] Maasz G, Zrinyi Z, Reglodi D, Petrovics D, Rivnyak A, Kiss T, Jungling A, Tamas A, Pirger Z (2017). Pituitary adenylate cyclase-activating polypeptide (PACAP) has a neuroprotective function in dopamine-based neurodegeneration in rat and snail Parkinsonian models. Dis. Model Mech..

[30] Noack C, Schroeder C, Heusser K, Lipp A (2014). Cardiovascular effects of levodopa in Parkinson’s disease. Parkinsonism Relat. Disord..

[31] Reichmann H, Lees A, Rocha JF, Magalhães D, Soares-da-Silva P, OPTIPARK Investigators (2020). Effectiveness and safety of opicapone in Parkinson's disease patients with motor fluctuations: the OPTIPARK open-label study. Transl. Neurodegener..

[32] Zeng BY, Balfour RH, Jackson MJ, Rose S, Jenner P (2010). Expression of catechol-O-methyltransferase in the brain and periphery of normal and MPTP-treated common marmosets. J. Neural. Transm..

