# Supplementary figures and images for: Cardiac tyrosine hydroxylase activation and MB-COMT in dyskinetic monkeys
Source: Sci Rep. 2021 Oct 6;11:19871. doi: 10.1038/s41598-021-99237-5 (PMC8494805; doi:10.1038/s41598-021-99237-5)

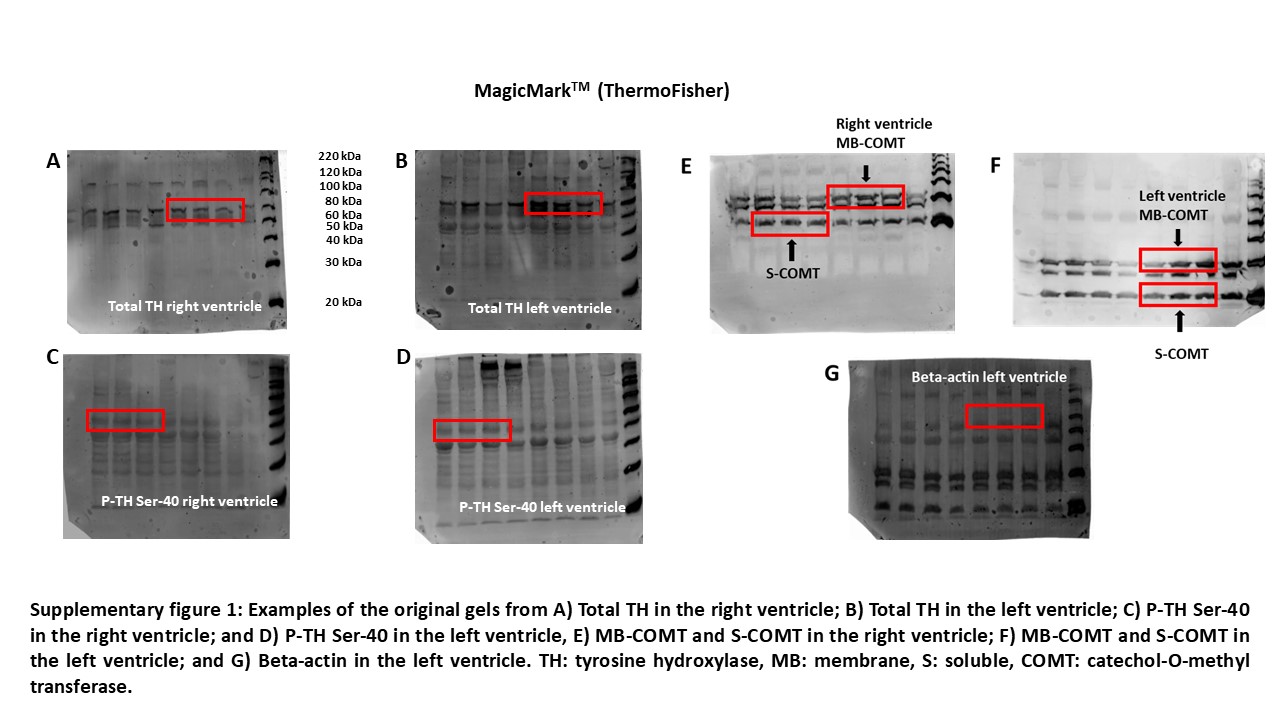

Supplement: Supplementary file 1 — Supplementary Figure 1. [file 41598_2021_99237_MOESM1_ESM.jpg]
